# Intraoperative Gamma Cameras: A Review of Development in the Last Decade and Future Outlook

**DOI:** 10.3390/jimaging9050102

**Published:** 2023-05-16

**Authors:** Andrew L. Farnworth, Sarah L. Bugby

**Affiliations:** Department of Physics, Loughborough University, Loughborough LE11 3TU, UK

**Keywords:** small gamma camera, SFOV gamma camera, portable gamma camera, intraoperative gamma camera, radioguided surgery

## Abstract

Portable gamma cameras suitable for intraoperative imaging are in active development and testing. These cameras utilise a range of collimation, detection, and readout architectures, each of which can have significant and interacting impacts on the performance of the system as a whole. In this review, we provide an analysis of intraoperative gamma camera development over the past decade. The designs and performance of 17 imaging systems are compared in depth. We discuss where recent technological developments have had the greatest impact, identify emerging technological and scientific requirements, and predict future research directions. This is a comprehensive review of the current and emerging state-of-the-art as more devices enter clinical practice.

## 1. Introduction

Radioguided surgery is a mature surgical practice that has seen considerable advancements in both technology and clinical applications over the past decade. Currently, most intraoperative guidance is non-imaging, where a surgeon uses a 1D gamma-sensitive probe to identify tissue-type-specific radiopharmaceutical uptake and to guide surgical decision-making [1,2].

**Figure 1:**
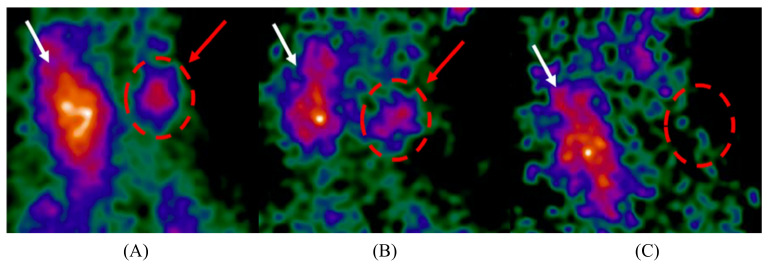
Intraoperative parathyroid scintigraphy images acquired using the Sentinella 102 intraoperative gamma camera. Radiopharmaceutical uptake can be seen in the thyroid gland (white arrow) and a hyperfunctioning parathyroid gland (red arrow and circle). Reproduced from Creighton et al. [4]. (**A**) Pre-incision image; (**B**) post-incision image; (**C**) post-excision image showing the removal of the hyperfunctioning parathyroid gland.

Small field-of-view gamma cameras, designed for intraoperative use, offer an increased benefit to surgical decision-making over gamma probes by visualising the radiopharmaceutical uptake within an anatomical region, shown in Figure 1. Intraoperative gamma cameras (IGCs) provide two key advantages over gamma-probes: the 2D field-of-view (FOV) allows a larger area of the surgical field to be surveyed in a single measurement, and the imaging provided is considered to be a more intuitive guide to decision-making than the numerical or audio output of a gamma probe [3].

**Figure 2:**
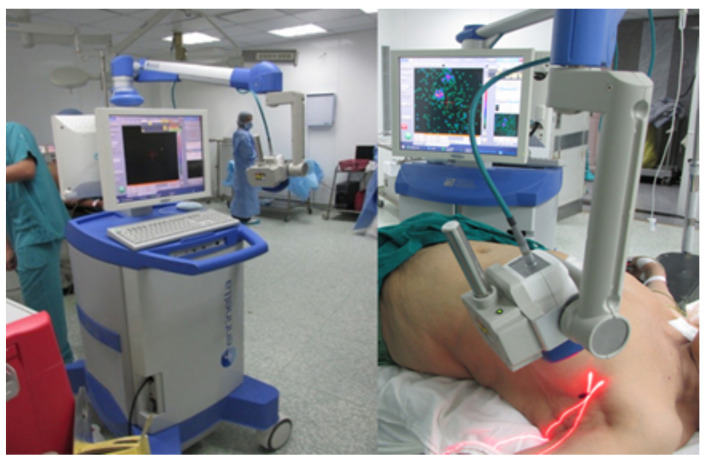
The Sentinella 102 intraoperative gamma camera. This arm-based gamma–optical device can project a laser cross-mark, placed at the centre of the gamma FOV, onto the imaging field. Reproduced from Ibraheem et al. [12].

The efficacy of an IGC for a radioguided surgical procedure is complex and depends on multiple aspects of the device’s design and how it is used. A device’s imaging performance, ease-of-use, level of integration within a surgical procedure, and the efficacy of the radiopharmaceutical protocol being visualised all contribute to the degree to which an IGC can aid a surgeon [3]. Consequently, when attempting to appraise IGCs, it is crucial to consider the design choices regarding the physical characteristics of a device alongside how the device is being implemented within the surgical environment. A current, commercial IGC being used within a surgical environment is shown in Figure 2.

The integration of commercial or research IGCs into radioguided surgery has been the subject of previous reviews [3,5,6] that comprehensively detail recent clinical experiences with a range of radioguidance devices. In contrast, the technological development of small-FOV IGCs has yet to be the focus of any dedicated review, although several authors have produced comparison tables for small- and large-FOV gamma cameras [2,7,8,9,10,11].

This work, building on the excellent foundation provided within Tsuchimochi and Hayama [2], provides a dedicated technical review of small-FOV IGCs, focusing on technological improvements and device properties. Following the methodology of Tsuchimochi and Hayama [2], this study has only included devices that are hand-held and are small enough to be used intraoperatively. All reviewed devices have been developed, updated, or have had new performance metrics published since 2013 and list radioguided surgery as an intended application.

## 2. Materials and Methods

A literature search was performed using relevant key words (e.g., “portable gamma camera” and “handheld nuclear imaging” in a range of permutations) to identify intraoperative gamma systems under development. This search was intentionally broad and resulted in a large number of papers that were manually sifted using the following inclusion criteria:

- Devices must be small and light enough to be operated whilst handheld, even if the device is intended to be used as part of an arm-based system.
- Devices must have a FOV suitable for intraoperative gamma imaging, identified as FOV sizes greater than 100 mm^2^.
- Intraoperative gamma imaging must be stated as an intended use case for the device.
- The characteristics of the device must have been published in a peer-reviewed journal or within technical documentation published by the device’s manufacturer.
- The device must have either been developed, undergone a technical update, or have had new technical information on the device published since 2013.

When an article was identified as relevant, the publications of its authors were reviewed to ensure the information provided here was up-to-date at the time of writing. The publications of authors cited by Tsuchimochi and Hayama [2] were also investigated to identify updates.

After publications meeting the inclusion criteria were identified, author names, institutions, the chronology of publications, and, in some cases, correspondence with authors were used to group publications by device. This allowed discussion of not only a snapshot of device performance but also a holistic view of the direction of development within the field over the last decade. Devices that have significantly branched in their development are discussed separately. For devices that are the subject of multiple publications, the performance characteristics of the most recent iteration of the device are used.

### 2.1. Choice of Parameters Reported

**Figure 3:**
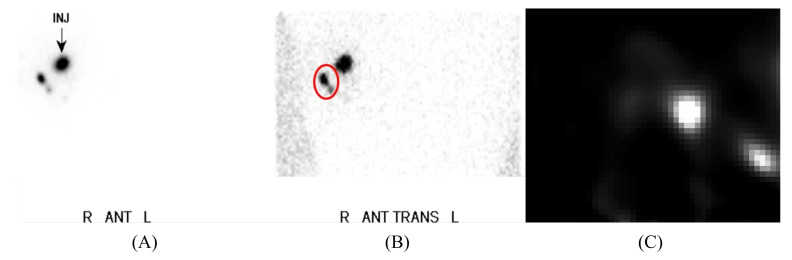
Pre-operative lymphoscintigraphy imaging, acquired using the KoglerCam. Reproduced from Kogler et al. [24]. (**A**) Anterior planar image indicating the injection site; (**B**) Right anterior oblique planar image, with uptake in two sentinel lymph nodes labelled in red; (**C**) A slice of a 3D fhSPECT acquisition corresponding to the red-circled area within image (**B**).

An effective IGC must have adequate spatial resolution to visualise lesions of interest, sufficient sensitivity to image without disrupting the flow of surgery, a large enough FOV to offer benefits over non-imaging probes, a suitable energy resolution to reject scattered photons, and be small and light enough to allow the operator to intuitively position the device. This wide range of necessary properties complicates device design, as the optimisation choices needed to maximise a single requirement typically reduce performance in others. Consequently, a balance between each of these requirements must be struck, where the degree to which one requirement may be sacrificed in favour of another is specific to the intended surgical application [13]. In addition to these basic requirements, some radioguided imaging techniques require additional device functionality, such as hybrid imaging capability [14]. Figure 3 shows an example of the breadth of imaging functionality that may achieved by an IGC within a single procedure, including both wide-field planar and 3D imaging.

A recurring theme throughout this review is the lack of standardisation in measurement parameters across the field. This makes direct comparison across devices challenging, as differences in experimental setups and calculation methods mean parameter comparisons were rarely like-for-like. Attempts have been made to introduce a standardised protocol for the performance characterisation of small-FOV gamma cameras [15], and it is important to acknowledge the effort made by multiple groups to address this issue by following specialised small-FOV standardised protocols or NEMA-based protocols [11,16,17]; however, the range of designs and intended applications of IGCs does somewhat negate a one-size-fits-all approach.

This lack of standardisation is particularly pronounced for extrinsic/system measurements, which are typically the measurements most relevant to clinical performance. As these parameters are distance-dependent, the rarity of any two groups reporting these values at the same distances actively hinders the usefulness of these measurements. These measures have been included due to their importance to the performance of effective IGCs, but great care should be taken when comparing values between devices. Extrinsic spatial resolution, extrinsic sensitivity, energy resolution, and FOV were the most commonly reported performance characteristics and so are reproduced here. These values are reported alongside the measurement distance except in cases where this has no effect (e.g., FOV for devices using parallel collimation geometries). In instances where a device has multiple collimators that produce different FOVs, a range has been stated.

**Figure 4:**
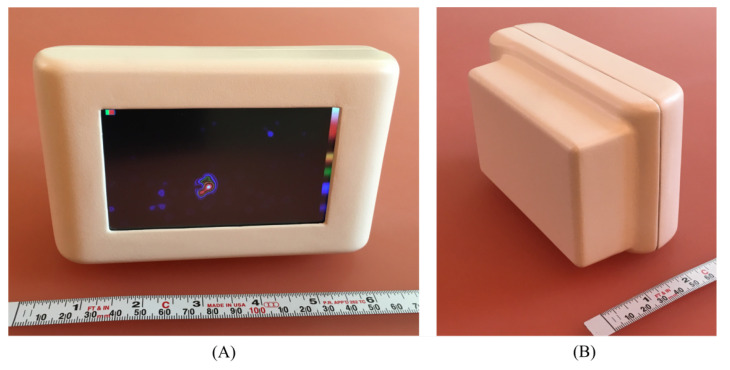
The ultra-portable PGC [31]. (**A**) User-side view of the device containing a display screen; (**B**) Object-side of the system—the rectangular area houses the device’s crystal–collimator structure.

Additional performance characteristics such as intrinsic spatial resolution, count rate capability, and non-uniformity have not been included due to the sparsity and incompatibility of data across systems. For example, only 7 out of 17 devices have published values for intrinsic spatial resolution [7,10,11,18,19,20,21], and for some device architectures, such as those using crystal–collimator structures (e.g., Figure 4), it may not be possible to directly measure this parameter. When reported, intrinsic resolution measures were found to be inconsistent between groups due to variations in both experimental setup and calculation method. Experimental techniques used to measure intrinsic spatial resolution were: estimation from extrinsic images of bar-type phantoms [7,20,21], deconvolution of collimated point-source profiles [7,18], and slit-type transition mask images [10,22,23]. The calculation methods to obtain intrinsic spatial resolution values were: the direct measurement of the full-width at half-maximum (FWHM) of intrinsic line spread functions (LSFs) [10,22], the deconvolution of the expected point-source profile shape from intrinsic images [7,18], quadrature subtraction of collimator geometric resolution from extrinsic LSF profiles [7], and FWHM measurement of the LSF obtained by taking the derivative of an edge response function (ERF) [23].

### 2.2. Calculations Used in System Comparison

For system measurements, the performance of a gamma camera is dominated by the design of its collimator [25]. To facilitate comparison between systems, the theoretical geometric efficiency and spatial resolution were calculated for each collimator geometry at specified distances (where this was possible based on published information) for 141 keV photons. These values are used in place of author-reported values to ensure methodological consistency.

Geometric efficiencies of parallel-hole collimators, $g_{parallel}$, were calculated by a commonly used formula [26]. This formula assumes that the aperture diameter is small compared to the aperture length.

(1) $$g_{parallel}=\frac{1}{4\pi l_{e}^{2}}\frac{A_{aperture}^{2}}{A_{unit}}$$

where $A_{aperture}$ refers to the area of a single aperture and $A_{unit}$ refers to the area of a single unit cell, including both the hole aperture and the septal thickness. The effective aperture length, $l_{e}=l-2\mu^{-1}$, approximates the collimator aperture length, *l*, experienced by photons considering imperfect attenuation; this is described by the linear attenuation coefficient, μ, of the collimator material [27].

Geometric spatial resolutions of parallel-hole collimators, $R_{parallel}$, were calculated to find the resolution at the detector-facing side of the collimator [25].

(2) $$R_{parallel}=\frac{d(l_{e}+h)}{l_{e}}$$

where *d* is the aperture diameter and *h* is the distance between the source and the object-face of the collimator. For dense collimator materials, which includes all collimators investigated within this work, the geometric resolution of a parallel-hole collimator at the collimator face is approximately equal to the aperture diameter. Consequently, geometric resolution values have not been stated at 0 cm distances to avoid unnecessary repetition.

On-axis geometric efficiencies for pinhole collimators, $g_{pinhole}$, were calculated considering penetration of the knife-edge of the pinhole aperture [28].

(3) $$g_{pinhole}=\frac{d^{2}}{16b^{2}}+\frac{\tan^{2}\frac{\alpha }{2}}{8b^{2}\mu^{2}}\;\cdot \;\left(1+\mu d\cot\frac{\alpha }{2}\right)$$

where *b* is the distance between the point source and the centre of the pinhole aperture and α is the full acceptance angle of the aperture.

On-axis geometric resolution values for pinhole collimators, $R_{pinhole}$, were calculated using formulae from Accorsi and Metzler, 2004 [29].

(4) $$R_{pinhole}=\frac{d_{res}\left(a+b\right)}{a}$$

where *a* is the distance between the centre of the pinhole aperture and the detector. The resolution effective diameter, $d_{res}$, adjusts the pinhole aperture diameter to correct for photon penetration of the knife-edge of the collimator and is given by:

(5) $$d_{res}=d-\frac{\ln\frac{1}{2}\tan\frac{\alpha }{2}}{\mu }$$

For collimators where the linear attenuation coefficient of the collimator material was not reported, values were calculated using data from the xraylib library [30].

It is necessary to emphasise that these theoretical calculations are approximations only and should be used carefully, particularly when comparing different types of collimators. However, the differences between actual and theoretical geometric parameters are significantly less than the difference that would be seen when comparing across different imaging distances.

## 3. Overview of Devices

This review identified 17 unique devices, of which 15 are new systems and 2 have been updated from the earlier versions investigated by Tsuchimochi and Hayama [2]. Two systems have seen no further development in the last 10 years. The MediPROBE device, which uses two readout architectures, has been reported as two devices in cases where the measures presented are affected by the readout used and a single device in other cases to avoid repetition. Where a particular clinical use case was identified, it was most commonly sentinel lymph node biopsy (SLNB), although the degree to which devices are focused for a single surgical application varies. Table 1 provides a summary of the devices that have been investigated.

### 3.1. Trends in System Functionality

One significant development since 2013 has been the integration of additional functionality alongside gamma imaging within portable devices. Multimodal imaging has now been realised in both research and commercial devices.

**Figure 5:**
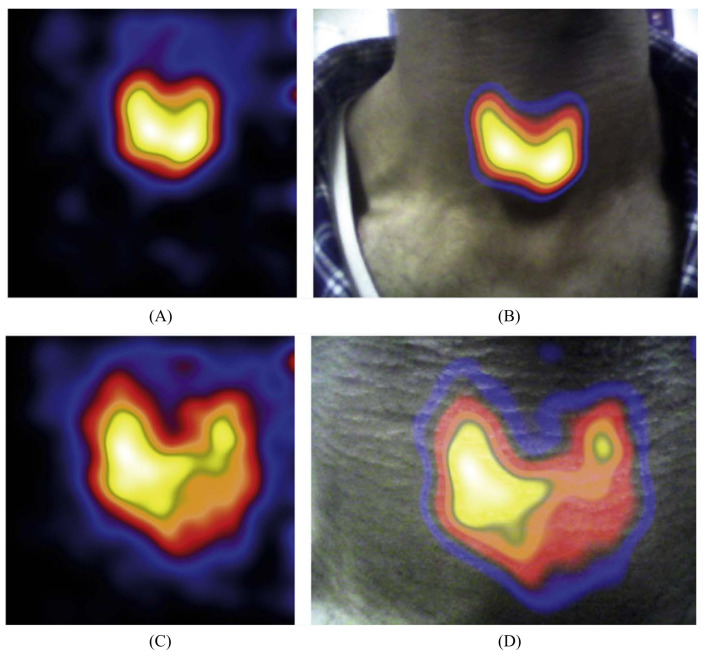
Non-surgical gamma and gamma–optical thyroid scintigraphy images acquired using the HCGC. Reproduced from Bugby et al. [23]. (**A**) Gamma-only thyroid image acquired at ~17 cm distance; (**B**) Combined gamma–optical image of the gamma-distribution shown in (**A**); (**C**) Gamma-only thyroid image acquired at ~8 cm distance; (**D**) Combined gamma–optical image of the gamma-distribution shown in (**C**).

Real-time hybrid gamma–optical imaging has been achieved by two systems. The HCGC uses a low-attenuation mirror positioned over the pinhole aperture to reflect optical light into an orthogonally orientated optical sensor, producing a matched imaging FOV and magnification over any imaging distance [78]. This has been demonstrated for both visible and NIR–fluorescence [71]. The Sentinella 102 places an optical module beside the gamma-imaging module, with images aligned through a calibration process [58]. Stereoscopic imaging, which provides distance information, has also been explored for the Sentinella 102 (optical only) and the HCGC (gamma–optical, including source depth estimation) [58,79]. Example gamma-optical multimodality images are shown in Figure 5.

Gamma–ultrasound multimodality imaging is also of interest due to the additional diagnostic information ultrasound (US) provides, e.g., cystic vs. non-cystic nodules, that are indistinguishable within gamma imaging [80]. A handheld gamma–US imaging platform has been developed by housing the CrystalCam and a separate US transducer within a 3D-printed casing [44]. The PolitoCam, which also targets gamma–ultrasound imaging, consists of a Hitachi linear ultrasound detector mounted in a transverse position in front of the collimator [21,80].

Multiple IGCs have been integrated within imaging platforms to provide intraoperative SPECT imaging and hybrid SPECT-US imaging. The declipse_®_SPECT system, which enables fhSPECT and US images to be fused, has seen extensive clinical use with the CrystalCam and KoglerCam [24,46,47].

Both 3D (SPECT) and gamma–optical imaging are typically utilised to improve localisation—the ability of a surgeon to orientate and locate sources within a patient. Two alternative localisation aids have also been developed. The Sentinella 102 features a laser pointing system allowing users to project a cross-mark onto the patient placed at the centre of the device’s FOV, as shown in Figure 2 [58]. The PGC integrates the display within the system itself, alongside power and processing, for untethered gamma imaging. This provides more perceptual feedback from the imaging process and more intuitive localisation [31].

Convenient mechanisms to vary collimation have also been developed, with multiple authors developing devices with interchangeable collimators or modular collimators with magnetic fixings [10,43]. The PopovicCam features a self-aligning, two-layer, modular, parallel-hole collimator with a magnetic fixing system made from a tungsten–polymer composite [10]. This allows users to quickly change the collimator aperture length, providing rapid adjustment of imaging spatial resolution and sensitivity [10,81]. The CrystalCam features interchangeable collimators that can be quickly changed without needing any specialised tools; they are designed for high resolution, high sensitivity, or medium energy imaging and are automatically detected by the system [43].

### 3.2. Trends in Physical Parameters

A wide range of weights of IGCs was found, ranging between 320 g–3.2 kg [8,10,11,24,38,43,52,56,64,76]. The majority of the weight of a camera head results from attenuating components, i.e., the collimator and detector shielding. Data relating to collimator weight and the degree of shielding (and associated leakage characterisation measurements) were poorly reported, leaving ambiguity in how device weight has been improved. The MediPROBE group provide a notable exception to this trend, clearly outlining the weight contributions of device components [8]. Future improvements in device weight are unlikely to be achieved by reducing shielding, as this is essential for camera performance; instead the optimisation of other components, such as cooling or electronics, is expected.

The size of IGCs also varied, ranging from 114 × 32 × 26 mm–103 × 211 mm [8,10,24,31,32,43,52,56,64,76]. Multimodal imaging devices or those with additional features, such as integrated screens, were found to have larger average volumes than gamma-only devices. This is to be expected due to the space required for additional components.

IGC FOVs ranged between 12 × 12 mm${}^{2}$–57 × 101 mm${}^{2}$ [9,10,18,20,21,24,31,32,38,43,52,57,64,76]. This a far broader range of FOVs than found in previous review works and is in keeping with the increased variation of intended device applications found. For example, some devices, such as the GoertzenCam, are intended to be used as visualising, probe-type detectors and consequently provide small FOVs, whilst other devices, such as the TReCam, have FOV maximisation as a key design priority for their intended surgical use-case [34,52]. This indicates that the development of device FOVs over the past decade has been effectively guided by application-dependent intraoperative imaging requirements [34]. The mean imaging area of the 13 IGCs that reported this parameter is 2341 mm${}^{2}$, which would equate to an FOV of ~48 × 48 mm for a square imaging field; this remains below the ideal FOV of 50 × 50 mm identified by Tsuchimochi and Hayama [2].

High device size and weight has been identified as restrictive to intraoperative mobility and can require devices to be mounted on an articulated arm to facilitate ergonomic use [3]. The application of industrial ergonomics guidance to orthopaedic surgery identified that the weight of handheld surgical tools should not exceed 2.3 kg in order to minimise work-related musculoskeletal injuries [82]. Of the 11 devices that reported a weight, only the MediPROBE exceeds the 2.3 kg limit; this is due to the weight of a 5 mm-thick removable lead shield [8]. Whilst this initially appears to suggest that a majority of reviewed devices are suitable for handheld use, blurring effects induced by hand-movement during image acquisition have been noted, particularly for imaging with a duration >60 s [83]. This indicates that arm-based platforms should be used for IGCs when attempting to optimise imaging performance, particularly for devices with poor handheld ergonomics and/or low sensitivities that dictate long acquisition times.

Unsurprisingly, the devices found to have the greatest FOV-to-volume and FOV-to-weight ratios were those that provide gamma imaging with no additional features. Given the noted importance of device size and weight for handling properties, this suggests a trade-off between device handling performance and additional imaging features that should be considered during the design of devices intended for handheld use. Consequently, the benefits of additional device features, such as multimodal or spectroscopic capabilities, should be weighed against a potential reduction in handheld imaging performance (in the context of the device’s intended surgical application).

Beyond device size, weight, and FOV, several groups have attempted to improve the physical characteristics of their devices by adding ergonomic features to aid handheld use [10,11,17,38,43]. The CrystalCam and PopovicCam should be noted in particular for displaying ergonomic features that are highly integrated within their device designs. The impact such ergonomic features have on surgical performance is currently relatively unexplored and is beyond the scope of this work.

### 3.3. Trends in Performance Characteristics

Reported energy resolution ranged from 5.2–58% at 141 keV [10,11,17,19,20,21,24,31,32,33,38,43,52,64,76]. Although the lower end of this range rivals the best-reported energy resolutions in Tsuchimochi and Hayama [2], overall there is a reduction in the proportion of IGCs that can be considered to have high energy resolution (<10% at 141 keV). This likely results from the (proportional) reduction in systems utilising semiconductor detectors in favour of scintillator detectors (although it may also indicate an increased focus on IGC design to applications where scatter is less influential).

Scintillator detector devices have reported extrinsic spatial resolutions of 1 mm @ 122 keV, 6.24 mm @ 140 keV, and 9.4 mm @ 140 keV at 1, 5, and 10 cm distances, respectively [17,20,52]. This represents an improvement in the best-achieved extrinsic spatial resolution at the camera surface since the previous review work, but is a reduction at further distances. The best-reported extrinsic spatial resolutions for semiconductor detector devices were 1.9 mm @ 140 keV, 1.09 mm @ 60 keV, and 8.2 mm @ 140 keV at 0, 5, and 10 cm distances, respectively [9,16,43]. These results indicate that both pinhole and parallel-hole collimator geometries allow for competitive extrinsic spatial resolution performance. Tests of coded apertures have also shown highly promising results, which are discussed in Section 4.5.

The maximum reported sensitivity was 1500 cps/MBq for scintillator detector devices and 554 cps/MBq for semiconductor detector devices, with both values measured at <50 mm distances [43,64]. Extrinsic sensitivity is a parameter particularly sensitive to changes in measurement distance. Given that sensitivity values have been reported at a large range of measurement distances, which were highly inconsistent between research groups, and in some cases were reported without a measurement distance, a more detailed comparison of extrinsic sensitivity between IGCs is not appropriate within this work.

## 4. Advances in Collimation Technology

**Figure 6:**
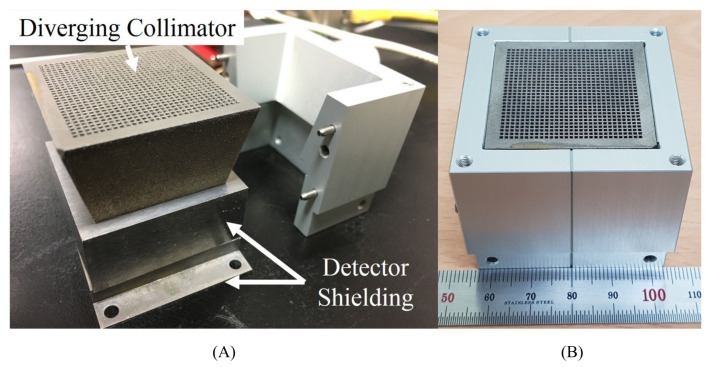
The 3D-printed, tungsten–carbide diverging collimator used within the JungCam. Reproduced from Jung et al. [76]. (**A**) Part-assembled view showing the diverging collimator and detector shielding; (**B**) Assembled view showing the collimator face within the exterior aluminium casing.

Collimator technologies used within IGCs have shown considerable advancement. Parallel-hole collimator geometries have continued to be the most used geometries, showing increasing design complexity throughout the time period of this work [10,17,18,21,32,38,43,52]. Multiple groups also chose to utilise alternative geometries such as diverging-hole collimators and knife-edge pinhole collimator designs [8,19,20,68,76]. The increased complexity of collimation geometries appears to have been enabled by advances in additive manufacturing techniques, such as 3D printing (e.g. Figure 6) and micro-casting [10,76]. Although only utilised by a single device, coded-aperture collimators have also continued to be an active area of research [9,77]. The degree to which collimators are integrated within the architecture of an IGC has also increased, with multiple groups utilising crystal–collimator structure architectures, which was originally implemented in the Imaging Probe device, where scintillator material is incorporated within the apertures of a parallel-hole collimator [31,64,84].

### 4.1. Collimator Material

No consensus was found for the most popular collimator material, with six devices utilising tungsten collimators [8,9,20,31,38,43,68,76] and six using lead collimators [17,18,21,22,32,64]. One device, the CrystalCam, used collimators made from both tungsten and lead [43].

Two novel collimator materials were found: a tungsten–polymer composite collimator, produced by a micro-casting process, and a tungsten–carbide collimator, produced by 3D printing [10,76]. Collimator material choice was highly reported, with only three devices omitting this parameter [18,20,52]. Monte Carlo simulation studies have established that tungsten collimators provide superior spatial resolution for 140 keV photons, and tungsten collimators are also likely to be less susceptible to mechanical damage than those made from lead [85]. Despite superior attenuation properties, tungsten has not dominated collimator material choice. This is likely due to the high cost of the raw material and the material’s combination of high melting point and mechanical hardness, which prevents complex part production via traditional manufacturing processes [86,87].

Additive manufacturing techniques, notably 3D printing by selective laser melting, appear to have overcome the traditional manufacturing limitations of tungsten and allow complex designs to be produced cost-effectively. Although additive manufacturing methods typically produce tungsten materials with a lower attenuation coefficient than the raw material, it has been shown that additively produced tungsten collimators retain their superior performance in comparison to lead collimators [87,88]. This represents an exciting area of development for IGCs as it allows both the improvement of existing collimator designs through the use of a superior material and makes possible new designs due to the increased complexity of collimators that can be manufactured.

### 4.2. Parallel Collimation

As in previous reviews, parallel-hole collimators remain the the most popular collimator geometry for IGCs and were found to be used in 11 out of 17 devices [10,17,18,21,24,31,32,38,43,52,64]. The collimator dimensions used and their associated geometric (calculated) performances are shown in Table 2.

The best geometric resolutions at 0, 5, and 10 cm distances were found to be 0.6, 3.6, and 6.6 mm, respectively [10]. The highest geometric efficiency identified was $2\times 10^{-3}$ [43]. As expected, the geometric resolutions of the parallel-hole collimators investigated are fundamentally limited by their hole diameters, and the fall-off of resolution with increasing distance is smallest for the collimators with the lowest aperture-diameter-to-hole-length ratios. Similarly, geometric resolutions were found to be inversely proportional to the geometric efficiencies, as expected.

### 4.3. Pinhole Collimation

Pinhole collimators have seen a slight reduction in popularity compared to previous reviews and are used by four devices [9,16,20,22,68]. The collimator dimensions used and their associated geometric (calculated) performances are shown in Table 3.

Pinhole collimators have achieved best geometric resolution values of 2.04 and 3.55 mm at 5 and 10 cm distances, respectively, and a maximum geometric sensitivity of $1.2\times 10^{-5}$ at 5 cm [9]. As expected, pinhole collimator geometries displayed superior geometric resolution values in comparison to parallel-hole collimators, but they typically showed geometric sensitivities at least one order of magnitude lower.

### 4.4. Collimator Optimisation

As noted above, the imaging characteristics of the majority of devices investigated are limited by their collimator properties. Consequently, efforts to improve the imaging performance of IGCs should focus on improving the suitability of the collimation method chosen for the intended imaging task.

Two key, non-exclusive approaches exist for the improvement of collimator performance. The first is to develop collimator geometries where the trade-off between geometric resolution and efficiency is less severe than in currently used collimation geometries. The second is to use in silico methods to optimise the design of currently understood collimation geometries. Here, an application-specific clinical imaging scenario is simulated for a prospective device geometry and captures the expected size and activity of the source anatomy. A series of images can then be produced, each using a different simulated collimator geometry. The image that produces the optimum balance of relevant image performance parameters is used to identify the best collimator design. This methodology has been used to optimise the collimator response of the λ-Eye device for SLNB [65]. This virtual prototyping of a range of designs prior to production is a time-efficient, cost-effective approach to improving device performance parameters when compared to repeated component fabrication and testing. This approach is limited by the computing power required and the need for the chosen collimation geometry to be well understood. Without knowledge of how changes to a collimator will affect image properties, it becomes impossible to use simulated images to guide design.

### 4.5. Alternative Collimator Geometries

Two devices use collimator geometries uncommon within IGCs. JungCam introduced the first use of a pixel-matched diverging-hole collimator, and MediPROBE introduced No-Two-Holes-Touching Modified Uniformly Redundant Array (NTHT MURA) coded-aperture collimators [9,76]. Given that image quality is predominantly limited by collimator performance and that conventional collimator geometries are well-optimised for intraoperative applications, novel collimation techniques represent a key development area for IGCs.

Table 4 provides the dimensions of the collimator used in the JungCam and Table 5 provides the properties of the coded-aperture collimators used by the MediPROBE device. Unlike for pinhole and parallel collimation geometries, the geometric performance of both diverging and coded-aperture collimators is less well understood and has additional complexity. Geometric performance parameters have not been included for these collimator types.

Coded-aperture collimators are a promising collimation technology that has yet to see widespread use within IGCs. These collimators use multiple pinhole apertures to achieve higher geometric sensitivities than single-aperture pinhole collimators whilst retaining their excellent geometric resolution and magnification properties. The many pinhole apertures project multiple, overlapping images onto the detector, which must be deconvolved to produce an image. Consequently, coded apertures are best suited to imaging objects that cover limited areas of the FOV. This reduces the complexity of image reconstruction and avoids high levels of ambiguity within the final image. SLNB has been identified as a suitable clinical application for coded-aperture collimators [93]. A further benefit of coded-aperture collimation is that the longitudinal depth of sources within an image can be estimated, as each superimposed projection that makes up the total image is acquired at a different sampling position [9,94], similar to stereoscopic imaging with conventional collimator geometries without the need for multiple images [79]. The addition of source-depth information to IGCs provided by coded-aperture collimators is notably valuable for intraoperative radioguidance due to the complexity of many surgical fields.

**Figure 7:**
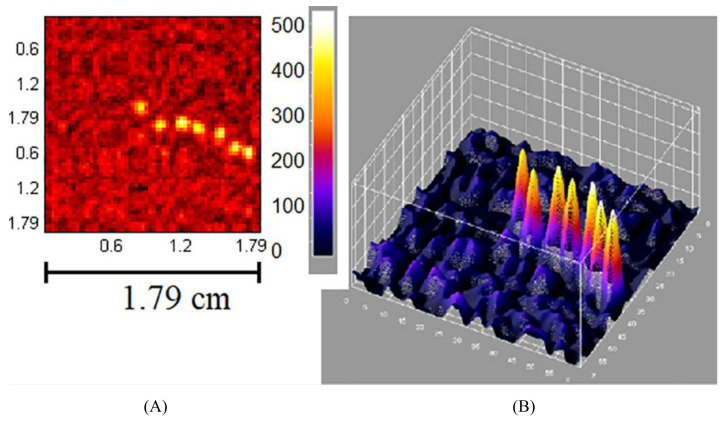
A demonstration of the high-contrast, real-time imaging potential of IGCs utilising coded-aperture collimators. Reproduced from Russo et al. [9]. (**A**) Imaging acquired by the MediPROBE device using a 0.08 mm aperture diameter, NTHT-MURA coded-aperture collimator. A 36 MBq ${}^{241}$Am source, located 49.3 mm from the collimator, was moved through seven positions during a 60 s-duration image; (**B**) 3D rendering of image (**A**). Note the excellent contrast and spatial resolution achieved in each of the ~8.6 s-duration dwell-times for each source position.

The initial use of coded-aperture collimators by an IGC appears to have been by the eZ-SCOPE device, which uses a pseudo-random-pattern array design to obtain source-depth information [95]. MURA-based coded-aperture designs, as used by the MediPROBE device, offer reduced noise in comparison to random-pattern arrays and have demonstrated significant improvements in image signal-to-noise measurements [96]. Modern applications of coded-aperture collimators to IGCs have yielded promising results. Russo et al. [9] used coded-aperture collimators to achieve a spatial resolution of 0.56 mm at 5 cm using a 60 keV point source and a longitudinal depth resolution of 3 mm, using a 27.5–35.5 keV ring-shaped source. This is by far the best extrinsic spatial resolution achieved by any device investigated within this review, although it should be noted that the current 0.08–0.11 mm collimator thickness appears to be insufficient for 141 keV photon imaging. These results imply that, provided the source geometry is suitable for coded-aperture imaging, spatial resolutions above all current collimator designs can be achieved and with higher geometric sensitivity than that of pinhole geometries. Given the current trend of devices specialised for specific surgical procedures, it seems likely that a coded-aperture-based IGC will achieve clinical imaging performance superior to all current devices in the future. This high performance is, however, expected to be associated with some loss of the source-object flexibility demonstrated by current IGCs. Figure 7 displays imaging representing the current state-of-the-art in IGC coded apeture collimation; achieving simultanious high resolution and high sensitivity performance.

## 5. Advances in Scintillator Detectors

Scintillator-detector-based IGCs have shown a large increase in popularity since the previous review work, being used in 14 out of 17 investigated devices [10,17,18,20,21,22,24,31,32,38,52,64,68,76]. Inorganic scintillator detector materials remain the most common detector technology for IGCs.

Scintillator materials new to IGCs, notably cerium-doped lanthanum bromide (LaBr${}_{3}$:Ce) and cerium-doped gadolinium aluminium gallium garnet (GAGG:Ce), were used in multiple devices [10,18,20,21,32,76]. Established scintillator materials, such as thallium-doped cesium iodide (CsI:Tl), have been applied using crystal structures novel to IGCs [68].

Scintillator readout electronics have also undergone considerable development, with large increases in semiconductor-based readout technology. This includes multi-pixel photon counter (MPPC) arrays of silicon photomultipliers (SiPM) and electron-multiplying charge-coupled devices (EMCCD) [31,52,68]. Devices using established readout technologies, such as position-sensitive photo-multiplier tubes (PS-PMT), have also progressed through the use of digital readout electronics [18].

### 5.1. Detector Size

The largest identified scintillator detector area was 57.2 × 101.4 mm, which is more than double that of any used by the devices within Tsuchimochi and Hayama [2,31]. The face areas of both continuous-crystal and pixelated-scintillator-array detectors has increased considerably, with pixelated devices showing slightly higher average detector areas (when excluding small, probe-like devices.) This increase in detector area appears to have been enabled by the implementation of MPPC readout arrays. Detector volume has also significantly increased. The KoglerCam displays the greatest detector volume found, with total detector array dimensions of 62.25 × 62.25 × 60 mm, achieved by using a crystal thickness 10 times greater than that of any other device [24].

### 5.2. Scintillator Material

Scintillator material choice has changed dramatically. The most-used scintillator material in earlier reviews, CsI:Na, has greatly reduced in popularity and is now only used by a single device [22]. Similarly, NaI:Tl, a traditionally popular scintillator material, is only used by one device [24]. In contrast, CsI:Tl, an established but previously uncommon scintillator material, has shown a large increase in popularity, with 6 out of 14 devices using the material [17,31,38,52,64].

Two scintillator materials novel to IGCs have been introduced: LaBr${}_{3}$, used by four devices [10,18,21,32], and GAGG:Ce, used by two devices [20,76]. This represents a shift towards scintillator materials with higher stopping powers, longer peak emission wavelengths, and improved light yields [97]. Counter-intuitively, the use of these improved scintillator materials has not necessarily resulted in improved detector performance. As the optical properties of the scintillator and the light collection properties of the detector geometry together determine the fraction of scintillation photons that may reach the photodetector, the use of a material with improved properties does not guarantee an increase in the number of detected scintillation photons and improved device performance [23,98,99]. To reap the benefits offered by these new materials, careful detector geometry design is needed.

### 5.3. Geometry

Scintillator detector architecture choice is not dominated by a single design, with five devices using continuous-crystal detectors [10,18,21,22,32] and seven using pixelated scintillator arrays [17,24,31,38,52,64,76]. Of the pixelated scintillator array devices, two use crystal–collimator structure architectures [31,64]. A clear link between scintillator material and detector geometry should be noted, with CsI:Tl, NaI:Tl, and GAGG:Ce predominantly used in pixelated geometries, and CsI:Na and LaBr${}_{3}$:Ce exclusively used in continuous-crystal geometries. This correlation likely stems from the practical considerations relating to the use of each material. This includes the manufacturing complexity and cost of a material, the need to seal hygroscopic materials, and the performance of a material for a specific crystal size [20,62,100].

**Figure 8:**
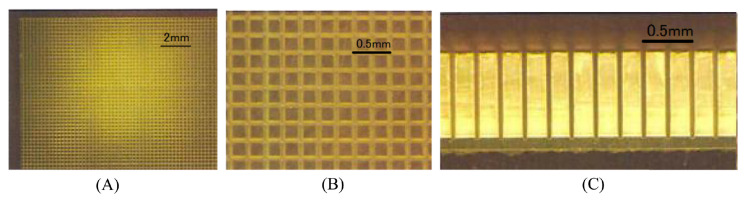
The grooved scintillator design implemented within the YamamotoCam [20]. (**A**) Object-side view of the grooved GAGG:Ce plate; (**B**) Magnified view of the object-side face; (**C**) Side view showing the 0.1 mm-thickness connecting material that makes up the continuous detector-side surface.

Two additional novel scintillator detector geometries were found: the micro-columnar-structure CsI:Tl detector of the HCGC, which constrains the spread of scintillation light by reflection of the optical photons down the needle-like column structures [68,101], and the ’grooved’ GaGG:Ce detector of the YamamotoCam (shown in Figure 8), formed by scoring a continuous-crystal with a dicing saw to produce a finely pixelated array on the object-facing surface and a continuous-crystal surface on the photodetector side [20,62].

#### 5.3.1. Pixelated Scintillators

Pixelated-scintillator-array detector geometries have increased in popularity. In 2013, pixelated geometries accounted for a third of IGCs; they now make up over half. This shift has been driven by a desire to improve spatial resolution by constraining the spread of scintillation light. The architecture properties of IGCs using pixelated-scintillator-array detectors are shown in Table 6.

As the intrinsic spatial resolution limit of a pixelated-detector device is given by the centre-to-centre crystal spacing, which may be smaller than the scintillation light-splash size for a given scintillator material and detector geometry, pixelation offers a method to increase device spatial resolution without requiring intra-light-splash-distribution event localisation [105]. This is convenient as, for small-FOV devices, the FOV fraction for which an event’s scintillation light-splash distribution will be truncated by the detector edge may be large. Truncated light splashes introduce positional offsets when traditional centre-of-gravity (COG) positioning algorithms are used, which reduces spatial resolution and degrades system energy resolution [99,106]. As more-robust positioning algorithms must consider truncation effects, more spatial information than that needed for COG algorithms is typically required. Pixelation offers an alternative method to increase device spatial resolution without increasing the complexity of readout signals. This is valuable, as low-complexity readouts allow signal multiplexing prior to analogue-to-digital conversion, which reduces device cost [102]. The pixelated design approach has lead to the development of finely pixelated detector arrays, exemplified by the JungCam, which uses a 29 × 29 array of 0.7 × 0.7 × 3.5 mm GaGG:Ce crystals, with each crystal wrapped in a BaSO${}_{4}$ reflective material to increase light-collection efficiency and reduce inter-crystal cross-talk [76].

#### 5.3.2. Continuous Scintillators

Although less popular, continuous-crystal scintillator detector geometries have also undergone development. The architecture properties of IGCs using continuous-crystal detectors are shown in Table 7. The continuous-crystal design approach is motivated by a desire to improve performance whilst avoiding the diminishing returns associated with reducing detector pixel size. This detrimental effect is due to the reflective media placed around each crystal element, which is needed to improve light collection efficiency and prevent cross-talk. As the elements of a pixelated detector array become smaller, reflective media contributes an increasing, insensitive fraction of the total detector volume. Decreasing pixel size therefore reduces sensitivity for a given detector volume. This issue is difficult to circumvent as, although detector depth can be increased to maintain a given active detector volume with pixelation, this, in turn, introduces a depth-of-interaction-dependent blurring effect for any photons incident at angles not orthogonal to the detector surface and increased scintillation light losses due to the self-attenuation of optical photons within the scintillator material. Consequently, reducing detector pixel size to increase spatial resolution only provides a benefit up to a certain point, after which the image-degrading effects of the small pixel size outweigh any benefits [107].

Optimisation studies of pixel size and inter-pixel reflective media width for SPECT applications have demonstrated this trade-off, with the best performance not necessarily arising from the smallest pixel size [108]. Continuous-crystal detector geometries avoid this trade-off entirely and are not limited by the pixel centre-to-centre spatial resolution limit, ultimately offering the promise of better spatial resolution than pixelated architectures. However, for this to be achieved, advanced event positioning algorithms are required, which consider the truncation effects noted above. This demands more complex data output and readout architectures, and more data-intensive devices [109,110]. The MAGICS device provides an excellent example of the continuous-crystal detector design approach, using a 51 × 51 × 5 mm LaBr${}_{3}$ detector coupled to an MPPC array with 256 readout channels. Event position is calculated by iteratively fitting a point-spread function model to the charge distribution data output by the SiPMs [32].

### 5.4. Readouts

Scintillator detector readouts have changed dramatically in the past 10 years. The rapid development of SiPM technology has allowed the use of MPPC readouts to become common within IGCs. This is unsurprising given the compact structure, mechanical durability, low voltage requirements, and insensitivity to magnetic fields that SiPMs demonstrate [111]. MPPC readouts are now used by 6 out of 14 scintillator detector devices, whereas no device used this technology in the previous review work [10,24,31,32,52]. Despite this fast uptake, PS-PMT readouts still remain slightly more popular, being used in seven devices [17,18,20,21,22,38,64]. Of the devices utilising PS-PMT readouts, five of the seven use multi-anode PS-PMTs [17,18,21,22,38], with the remaining two devices using cross-plate-anode-type PS-PMTs [20,64]. A single device was found using a readout technology novel to IGCs, the HCGC, which uses a highly pixelated EMCCD readout; notably, this readout technology provides by far the smallest readout pixel size found in any device and reports no inter-pixel dead space [68]. Across all readout technologies, a significant trend towards pixelated readout technologies has been found.

The largest readout area found was 58.4 × 102.2 mm, achieved by using a 4 × 7 array of three-side abuttable MPPC boards, by the PGC [31]. This is a readout area 2.5 times larger than that achieved by PS-PMT readout scintillator devices and 4 times larger than that achieved by semiconductor devices [17,21,22,38,43].

The recent, rapid uptake of MPPC readouts indicates this will become the dominant IGC readout technology in the near future. This advancement has been achieved by moving from arrays of individual SiPMs, or small prefabricated MPPC boards that could not be tessellated, to prefabricated, abuttable MPPC boards that can be used to form large arrays. Importantly, this has both increased the total readout area possible to achieve using SiPM-based readouts and reduced the inter-SiPM dead space.

The reduction in MPPC inter-SiPM dead space is also an important development for this technology. Unlike segmented-anode semiconductor or MA-PMT detectors, for which charge-carriers moving throughout their volume induce a readout signal, inter-SiPM space in MPPCs is entirely insensitive to ionization events. Incomplete MPPC fill-factors therefore reduce device sensitivity if the width of the dead space is large enough to obscure inter-SiPM scintillation light-splashes. This effect can be offset by careful detector geometry design and scintillator material choice, or by simply reducing the dead space to below the expected light-splash distribution width. The GeortzenCam showed the highest MPPC fill-factor, achieved by using a single MPPC board to produce a small active readout area with minimal insensitive area [52]. Multiple subsequent devices have made use of abuttable MPPC boards to achieve active readout areas 10 times larger than that of the GeortzenCam, with only a moderate sacrifice to the readout active fraction [31,32,76].

## 6. Advances in Semiconductor Detectors

Semiconductor detector IGCs have continued to centre around the use of CdTe and CdZnTe detector materials, with development focusing on the size of detector crystals used and the complexity of application-specific integrated circuit (ASIC) readout electronics [7,9,43]. All current semiconductor detector devices utilise the hybrid pixel detector device architecture, which combines a continuous-crystal detector with pixelated anode contacts and a miniaturised complementary metal–oxide semiconductor (CMOS) ASIC readout. This two-component approach allows the simultaneous use of both a high-Z semiconductor suitable for gamma imaging and silicon-based readout electronics [112]. Importantly, as this architecture allows each of the pixelated anode contacts to be connected to its own readout circuit, the readout ASIC used determines how the charge at each pixel is sampled and the readout complexity. The flexibility of this two-component design approach has allowed advanced, general purpose readout ASICs to be developed that may be used with a wide range of detector materials and in a wide range of device geometries [113]. The application of these advanced ASICs to IGCs has greatly increased both the complexity of data that can be read out per pixel and readout timing resolution [9]. This has allowed IGCs to achieve spectroscopic imaging, where the charge readout is subject to multiple thresholds that allow the energy of each event in each pixel to be quantified. This is exemplified by the CrystalCam device, which features 4095 energy channels per pixel, allowing a pixel-specific energy spectra to be produced from any image [43]. In addition to their impressive readout performance, semiconductor detector IGCs have achieved the current state-of-the-art values for both extrinsic spatial resolution and energy resolution [9,43].

Semiconductor detector IGCs have shown a reduction in popularity since the previous review, with only three devices under current development [7,9,43]. As two of the investigated devices represent varying stages of the iterative development of the MediPROBE device, only a single new semiconductor detector IGC was found. This indicates a drastic reduction in the number of research groups developing these devices.

Table 8 summarises the semiconductor detectors used within current IGCs.

### 6.1. Detector Size and Geometry

Semiconductor detector size has increased, although both average detector areas and volumes still lie below those achieved by scintillator detector devices. Whilst some earlier semiconductor detector IGCs utilised pixelated detector arrays, all current systems use continuous-crystal detector geometries with monolithic cathode contacts and pixelated anode contacts [7,9,43]. The CrystalCam utilises the largest single-crystal semiconductor detector implemented within an IGC, with dimensions of 39 × 39 × 5 mm [43].

### 6.2. Detector Material

Semiconductor detector materials remain unchanged over the past decade, with two devices found using CdTe:Cl [7,9] and a single device using CdZnTe [43]. Although familiar detector materials have been reported, it is unclear whether the radiation detection properties of these materials have also remained constant, as material compositions, contact types, and contact materials are historically poorly reported. Given the importance of these parameters in determining the charge transport properties of a detector, the omission of this data prevents any meaningful comparison of semiconductor detector materials used within IGCs [115].

### 6.3. Detector Architecture

Semiconductor detectors face similar geometric trade-offs as those affecting pixelated scintillator arrays. To achieve suitable performance, detector thickness and anode pixel size must be carefully optimised.

Increasing detector thickness increases the fraction of incident gamma rays that are attenuated and improves detector sensitivity. However, in non-ideal materials, the probability of charge carrier trapping and recombination effects, caused by crystal defects and impurities, increases with longer charge carrier path lengths. Any charge loss due to these effects degrades detector energy resolution. This creates a detector-thickness related trade-off between sensitivity and energy resolution that must be managed by careful detector design.

A similar trade-off is seen with anode pixel size. Reducing anode size typically improves intrinsic spatial resolution and reduces the impact of imperfect charge carrier transport [109,116,117]. However, should the anode size become comparable to the charge carrier distribution width, then charge sharing will occur, where a single event will register across multiple pixels. This introduces charge loss within the inter-pixel region and creates an anode-size-related trade-off between intrinsic spatial resolution and energy resolution. Anode pixel size should therefore be optimised for the spatial and spectral resolution requirements of the intended application. It is important to note that the regular nature of charge sharing allows the use of correction algorithms to partially recover energy resolution [118], or the application of centroiding algorithms to achieve sub-pixel spatial resolutions [8,119]. These algorithms have yet to be implemented within an IGC but offer the potential to improve performance in the future.

The architecture choices made in balancing these trade-offs largely determine the behaviour of the semiconductor detectors investigated within this review, although the magnitude of this impact is, in part, due to the limited range of detector materials used. When considered alongside the readout capabilities of the ASIC used, it becomes possible to understand the design choices made by the development groups.

For example, the Medipix2 MediPROBE device uses highly pixelated anode detectors that provide excellent intrinsic spatial resolution at the cost of charge sharing. As 140 keV photon events cover an average of 2.27 pixels, each event-pixel will register a fraction of the total charge. This prevents traditional energy windowing. Instead, short-exposure frames are used to image the multi-pixel charge clusters, and cluster centroid location is used to determine event position [8]. Whilst the two energy thresholds implemented within the Medipix2 ASIC do not provide sufficient granularity to quantify the energy of the fractional charges, which is required for advanced charge-sharing correction algorithms, this approach allows intrinsic spatial resolution to be recovered despite significant charge sharing. This represents a design choice to maximise spatial resolution performance of the MediPROBE, given the limitations of the Medipix2 readout ASIC used, at the cost of event energy discrimination.

In contrast, the CrystalCam utilises anode sizes comparatively larger than those used by the MediPROBE devices. This detector architecture provides excellent energy resolution, as the likelihood of charge sharing is reduced, especially when considering the threshold-based trigger mechanism for pixel readout, which is unlikely to be activated by small-magnitude charge-sharing signals [48]. As only a single pixel is likely to trigger for any event and no sub-pixel signal localization method is used, the intrinsic spatial resolution of this device is limited by the anode pixel pad size. This represents a design choice to prioritise energy resolution over spatial resolution, which was likely motivated by a desire to take advantage of the excellent spectroscopic readout properties of the XAIM ASIC.

### 6.4. Readouts

Semiconductor detector readout ASICs have undergone considerable development, which has focused on increasing the functionality of pixel readout, to provide more detailed readout data per detected event, and to increase readout timing resolution.

**Figure 9:**
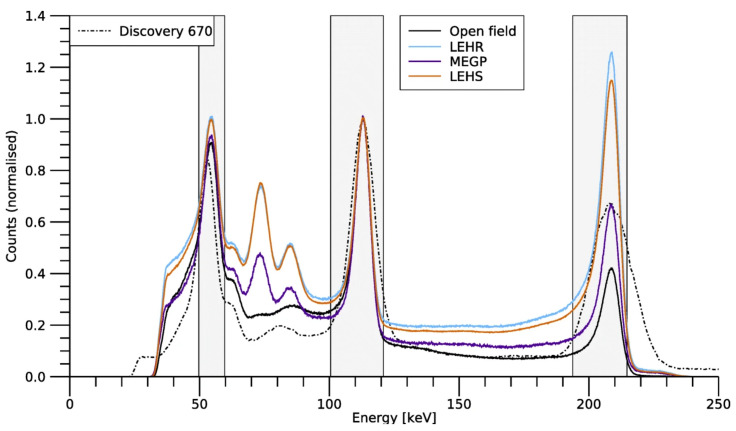
${}^{177}$Lu spectra acquired by the CrystalCam for each of the devices collimation options: open field, which indicates an intrinsic measurement; LEHR; MEGP; and LEHS. In comparison to a conventional GE Discovery 670 gamma camera, this semiconductor-detector IGC achieves superior energy resolution across a broad energy range. Count values have been normalised to unity at the 113 keV peak. Reproduced from Roth et al. [45].

The CrystalCam device is based on the single-photon-counting, 128-channel, XAIM readout ASIC, featuring user-programmable trigger thresholds and signal calibration functionality for each pixel. When triggered, this ASIC provides readout of a trigger signal, pixel channel number, and the amplified event signal [48]. This ASIC does not support multi-pixel readout, as only a single peak pixel value is recorded during multi-pixel events [120]. The amplified event signal is then digitized within the detector module to give a 0–4095 energy channel value that is output as list-mode data alongside a timestamp and pixel number [43,45]. The implementation of the XAIM ASIC within the CrystalCam represents the first time spectroscopic imaging has been achieved by an IGC. The excellent, whole-detector energy resolution achieved by the CrystalCam is shown in Figure 9.

The MediPROBE device has predominately utilised the Medipix2 readout ASIC [7,8,9,16]. This single-photon counting ASIC provides 256×256 identical readout pixels, which can be individually calibrated. Amplified detector signals are subjected to two independent, user-defined threshold values providing event energy windowing. If a signal lies between these threshold values, the pixel’s shift register acts as an on-pixel 13-bit counter, with an 8001 count dynamic range, and records the event. A frame-based readout, where a raised ‘shutter’ voltage supplied to the shift register pauses data acquisition, allows the pixel data to be readout in either serial or parallel modes. This achieves frame read speeds of 9 ms for serial readout and 266 μs for parallel readout for an external clock speed of 100 MHz [121]. The combination of this fast frame read speed and the low radiation fluences experienced during scintigraphy allows the MediPROBE to reliably capture low-occupancy image frames. This greatly reduces the likelihood that multiple charge-sharing clusters within a single frame will overlap and introduce event positioning errors. Consequently, the fast readout provided by the Medipix2 ASIC acts to improve the spatial resolution of the MediPROBE.

The latest versions of the MediPROBE device have featured the Timepix ASIC, which was designed to simultaneously provide precise event timing and quantification of the charge deposited. As an iterative development of the Medipix2 ASIC, the Timepix ASIC employs a similar operation method, with two key differences [9,114]. All pixels now use a 14-bit shift register, with a dynamic range of 11,810, and the ASIC may now be operated in three modes. The three acquisition modes are: counting mode, where each pixel operates in the same manner as Medipix2; time-over-threshold (TOT) mode, where the counter is continuously incremented whilst the preamplifier output remains above the threshold value; and arrival time mode, where the counter runs from initial triggering until the shutter mode is set to readout. Importantly, as the decay time of the preamplifier signal is proportional to the measured charge, TOT mode provides a method of quantifying event energy on a per-pixel basis, as required to perform spectroscopic imaging [114]. Current implementations of the Timepix ASIC within the MediPROBE device do not appear to have implemented this functionality [9].

## 7. Outlook for the Next 10 Years

Collimator performance dominates current IGC capabilities; this, therefore, represents a research area with a high potential to improve overall performance. Whilst pinhole and parallel-hole collimator geometries are well-established and understood, diverging and coded-aperture designs are still in flux, with active research into both collimator design and image reconstruction [122,123,124]. This development is supported by the rapid progression of additive manufacturing for high-Z materials, although currently cost remains a barrier to this technology’s uptake. As prices come down and complex geometries become more understood, developers of IGCs are expected to capitalise on the capabilities of this manufacturing technique to create smaller, more complicated, non-traditional collimator designs. This effect is already being seen at the early design stage and looks set to considerably impact the range of collimation geometries used in the future [122,125]. Multiple research groups are also currently seeking to develop detector geometries that are not collimator reliant, although these devices remain in the early stages of development [126,127,128,129].

New scintillator detector materials continue to be identified, and the development of structured scintillators will enable tuneable scintillator properties [130,131,132]. This highly active area of research looks set to allow scintillators to be precisely optimised both for the radiation detection task and readout system compatibility.

Semiconductor detector materials also continue to be an area of high research interest, with the CdTe and CdZnTe family of materials continuing to be developed. The addition of selenium to the CdZnTe matrix offers improved crystal growth properties whilst avoiding the intrinsic and lattice defect limitations of current CdZnTe. This new material, ‘CZTS’, may supersede current CdZnTe detector performance and be less costly to produce [133,134]. In addition, entirely new semiconductor materials are also under development. TlBr is a promising room-temperature semiconductor material that has been researched for several decades. This material’s low melting temperature and cubic structure makes it suitable for large, high-quality crystal growth, and its high relative atomic number and density provide a higher stopping power than CdTe or CdZnTe. Although current TlBr detectors show energy resolutions below that of CdTe or CdZnTe, the charge-carrier transport properties of TlBr continue to improve, and it may be that this material can provide superior radiation detection properties in the future [135]. Finally, metal halide perovskites appear promising for the development of both semiconductor and scintillator detector materials [136].

Readout technologies for both scintillator and semiconductor detectors continue to show significant development. The rapid-uptake MPPC photodetectors shown in this work look set to continue, and MPPCs are highly likely to become the dominant semiconductor readout technology in the near future, especially given that the development trend for SiPM technology demonstrated over the past decade, with dramatic increases seen in sensor density, fill factors, and photon detection efficiency, looks set to continue. Large-format, high performance, CMOS-based MPPCs are expected to become available in the medium term [137]. Scintillator detectors using frame-based readouts look to be able to exploit the rapid development of scientific CMOS photodetectors, which promise faster readout speeds and lower noise levels than conventional CCD-based photodetectors [138]. Semiconductor detector devices also look set to see considerable readout development, with a huge range of hybrid pixel detector ASICs currently available, many of which are not currently applied to IGCs. These new ASICs offer advanced readout capabilities, such as exceptional temporal resolution, improved active areas, spectroscopic imaging capabilities, and on-chip charge-sharing correction [139,140,141].

There has been a notable trend in recent publications to investigate how different camera parameters interact with one another, e.g., collimator material and geometry [142], collimator and scintillator pixel sizes [143], scintillator materials [99,101] and structure [144], and anode and readout geometry [145,146]. This represents a change in the focus of IGC development from the past, where individual component optimisation was prioritised, to a more holistic design approach considering the total performance of the system at a design stage. In particular, it appears likely that future intraoperative devices will use Monte Carlo methods to optimise device performance parameters for a specified intraoperative application. Whilst it is clear from this work that there is no one-size-fits-all perfect combination of design parameters, future devices will be able to leverage the increased understanding of camera component interactions to achieve even more specialised, high-performance devices. We are already seeing this, albeit at an early stage, with many of the devices investigated here being designed specifically for certain procedures (for example, the SNLB-specific design of the λ-eye device), and this trend looks firmly set to continue.

To date, advances in IGCs have been achieved by improvements in component hardware, such as increasing the quantum efficiency of a PMT photocathode by using higher purity materials, and improvements in event-positioning algorithms. Future advances in performance look to also be achieved through advanced data processing methods. Super resolution techniques, which use multiple low-resolution images to reconstruct a single, high-resolution image, do not rely on improving a device’s intrinsic or collimator performance to achieve increased spatial resolution, therefore offering a route by which the current spatial resolution limits, introduced through the use of conventional collimation geometries, can be overcome [107]. Charge-sharing correction algorithms, which have already been implemented for multiple detector systems, offer the ability to recover the spectral performance lost by small-anode-pixel semiconductor detectors and allow for exceptional energy resolution and spatial resolution simultaneously [118,147,148]. The application of deep learning and neural networks to the optimisation of current data-processing tasks looks to advance IGCs in a range of ways, including: improved energy resolution reconstruction, sub-pixel event positioning, improved event localisation, and improved near-field coded-aperture image reconstruction [119,124,149,150].

As noted by Tsuchimochi and Hayama [2] 10 years ago, the lack of testing and reporting standardisation makes it very difficult to compare devices. This has not changed, and the complexity of comparing different system designs limits progress of the field as a whole. The benefit of standardised metrics is for comparison, but if they are not collected using standardised protocols, this is lost.

The vast range in designs—making some traditional parameters inapplicable or impossible to measure—and the trend towards hyper-application-specific design, which renders some parameters irrelevant when aiming to assess practical performance, suggests that a single-checklist protocol is not the best approach. Instead, we expect to see more publications using simulated clinical scenarios, focusing on only the characteristics relevant to the application of choice. We would hope that some of these scenarios, SLNB for example, are sufficiently common that the community will reach a consensus on appropriate experimental setups and imaging parameter requirements. We can all do our part by, when possible, using common stand-off distances for extrinsic measurements (e.g., 3 cm, 5 cm, and 10 cm) even if only in addition to the distances most-suitable for a particular device.

Radioguided surgery is currently an established practice and has been used for a large range of surgical procedures. As the availability and range of tracers is continually developing to meet the clinical need for receptor-specific radiopharmaceuticals, the scope of radioguided surgery is also expected to broaden. This increased range of applications will likely further the already noted trend for multi-modal intraoperative imaging systems and multi-modal tracer development. As already demonstrated by current multi-modality procedures, these devices will allow surgeons to leverage the power of multiple disease-specific markers simultaneously. This is expected to increase the overall efficacy of intraoperative imaging tools [44]. Given the important ability of gamma-detection to localise deep lesions, radioguided surgery—particularly image-based radioguidance—appears to be a key technology to support the expansion of minimally invasive surgery in the future [3]. However, for this to be realised IGCs must successfully transition from lab-based research devices into the clinical setting. Historically, the number of devices that have successfully made this transition has been very small, despite a clear need for improved intraoperative tools. This remains a key challenge facing the IGC community which must be addressed for this technology to reach its potential.

The expansion of IGC applications is also expected to be driven by the continued development of IGC energy-resolution performance and spectroscopic capabilities. Future devices are expected to have sufficient energy resolution to perform techniques that are beyond the current capabilities of IGCs, notably multi-isotope and therapeutic radionuclide imaging.

Multi-isotope imaging, which refers to the simultaneous imaging of multiple radiopharmaceuticals each labelled with a different radioisotope, offers the potential to study multiple physiological processes in tandem. This may improve the diagnostic information obtained by an image by providing a more complete understanding of the patient’s physiology. This is of particular relevance to the intraoperative imaging environment where, unlike imaging within a conventional nuclear medicine department, a patient cannot be rescheduled for further imaging following the biological clearance of the radiopharmaceutical should the information gained from a single-isotope study be insufficient to inform surgical decision-making. Although, to date, multi-isotope techniques have been developed for a large range of clinical applications [151], the energy resolution of current gamma cameras has limited the success of clinical dual-isotope imaging [152]. The excellent spectral properties of the next generation of IGCs is expected to allow multi-isotope imaging without the degradation of image quality due to cross-talk between differing isotopes’ spectra. Although not yet demonstrated intraoperatively, the advent of multi-isotope imaging appears to be near from a technological standpoint, having already been achieved by small-FOV gamma cameras [153,154,155,156], intraoperative gamma probes [157], and within small-animal imaging [158].

Alongside the continued development of low-energy-gamma-emitting radionuclide tracers, recent trends in radioguided surgery and IGC development have seen an increase in the use of beta-emitting isotopes and those traditionally used for therapeutic applications [3,45], such as the use of ${}^{125}$I seed sources as tumour markers during radioactive occult lesion localization (ROLL) procedures. By implanting a focal, sealed source within the tumour, this technique can aid localisation in cases where either no suitable tracer for radioguidance exists, or poor/diffuse uptake/retention of liquid tracers would render traditional radioguidance techniques ineffective [159]. As the gamma-photon energies emitted by therapeutic isotopes typically provide suboptimal imaging performance when imaged by gamma cameras intended for diagnostic radionuclide imaging, this area appears to be an ideal candidate for the development of application-specific devices.

## 8. Conclusions

IGCs have seen a dramatic expansion of research interest and a high pace of development—excitingly, these trends look firmly set to continue. In addition to the multiple research groups continuing to develop the devices identified within this work, many research groups are also active in the early development stages of both IGCs and the innovative technologies required for their future development [126,160,161,162,163,164]. A decade ago, Tsuchimochi and Hayama [2] astutely noted the potential that the development of IGCs held for the surgical environment and the advancement possible due to both their technological advancement and the continuing expansion of nuclear medicine and molecular imaging. Their message has been proven to be true and looks to remain true for another, upcoming decade. The broad range of technologies under development and the huge quantity of novel ideas being investigated show clear promise for IGC research. Given the ever-expanding scope of radiopharmaceutical development to target an increasing range of pathologies and the ability of highly application-specific IGC design to produce specialised surgical tools, it is expected that intraoperative, image-based radioguidance techniques will see application in an increasing range of surgical procedures in the near future.

## Figures and Tables

**Table 1:** A summary of the intraoperative gamma cameras identified in this review and their architectures, ordered by the date of the last update. Devices that are not named within publications have been named after the first author of their key paper. Bold keywords in the description column indicate additional functionality beyond gamma imaging. The precision of measurements is reproduced from published work and is not consistent across devices. Approximate values (indicated by ~ signs) are reproduced from published works. All performance characteristics are for 141 keV photons unless otherwise stated. Device sizes are reported as width × height × length as measured from the imaging face of the device, and FOV values are reported in width × height format. Footnotes appearing at the bottom of the table are necessary due to the range of reported data.

Device	Design				Performance Characteristics			Physical Parameters		
	Description	Detector	Readout	Collimator	Extrinsic Spatial Resolution (mm)	Extrinsic Sensitivity (cps/MBq)	Energy Resolution (%)	Size (mm)	Weight (kg)	FOV (mm)
TReCam [18,32,33,34,35,36,37]	Developed in 2009 for tumour resection applications, with new device information published in 2015. Designed to achieve a larger FOV than the POCI device, as requested by surgical feedback. Used intraoperatively for SLNB.	LaBr${}_{3}$ scintillator and a Hamamatsu H9500 flat-panel 256-anode photomultiplier tube (MA-PMT).	Four HARDROC2 semi-digital readout ASICs. Pulse centroid position obtained using a power-weighted, centre-of-gravity (COG) algorithm.	Parallel (LEHR)	2 @ 0 cm	300 @ 0 cm	11	83×83×117	-	50×50
IPG 2 [38,39,40,41,42]	Commercialised IGC. Used intraoperatively in SLNB and parathyroidectomy procedures.	Pixelated CsI:Tl scintillator array and a Hamamatsu H8500 flat-panel MA-PMT.	Custom USB ADC-card. The software interface returns no spectral information.	Parallel (LEGP)	2.5 @ 0 cm 2.9 @ 1.5 cm	204 @ 0 cm	20 ^a^	70×70×270	1.2	44.1×44.1
CrystalCam [43,44,45,46,47,48,49]	**Spectroscopic** Commercialised device featuring per-pixel spectroscopic capability and suitable for ${}^{177}$Lu imaging. Integrated within multiple multimodal imaging platforms. Used intraoperatively in SLNB.	Indium-contacted Cd${}_{0.9}$Zn${}_{0.1}$Te crystal grown via the modified horizontal Bridgman technique.	Two XAIM readout ASICs capable of per-pixel, 12-bit, spectroscopic imaging.	Parallel (LEHR)	1.98 @ 0 cm ^b^ 4.9 @ 5 cm ^b^	237 @ 0 cm	5.2	65×65×180	0.8	40×40
				Parallel (LEHS)	2.63 @ 0 cm ^b^	554 @ 0 cm				
				Parallel (MEGP)	1.90 @ 0 cm ^b^	177 @ 0 cm				
PopovicCam [10,50,51]	Designed to provide a small, lightweight device for handheld use, based on requirements outlined by melanoma surgeons. Used preoperatively and intraoperatively for SLNB.	LaBr${}_{3}$ scintillator and an MPPC consisting of 80 SiPMs arranged in a grid pattern approximating a circle.	All 80 SiPM channels are digitised and read out. Event positioning is by a COG algorithm.	Modular parallel (×1)	7.5 @ 3 cm 10.3 @ 5 cm 16.5 @ 10 cm ^c^	481 @ 0.3 cm	21.1	⌀ 75 × 40.5	1.4	⌀60
				Modular parallel (×2)	4.5 @ 3 cm ^c^ 6.5 @ 5 cm ^c^ 9.5 @ 10 cm ^c^	73 @ 0.3 cm				
GoertzenCam [52]	Designed to be used in place of non-imaging gamma probes in SLNB procedures. This is the smallest and lightest device investigated.	Pixelated CsI:Tl scintillator array and a SensL SPMArray4 SiPM.	Analogue SiPM signals digitized by two-channel analogue-to-digital converter (ADC) before 8:1 multiplexing	Parallel (LEHR)	3.46 @ 0.1 cm @ 122 keV 6.24 @ 5 cm @ 122 keV	162.9 @ 0.1 cm @ 122 keV 149.7 @ 5 cm @ 122 keV	38.9	32×26×114	0.32	13.2×13.2
MAGICS [32,53,54]	Developed to address the size and weight limitations of available devices. The small size of MAGICS was achieved using miniaturised readout electronics.	LaBr${}_{3}$ scintillator and an MPPC array of 4 Hamamatsu S11828-3344M MPPCs.	Four EASIROC ASICs provide analogue readout of the 256 channels before digitisation.	Parallel	2 @ 0 cm ^e^	300	9.78 @ 122 keV	83×83×83.5	-	51×51
Sentinella 102 [4,22,42,55,56,57,58,59,60,61]	**Hybrid; Localisation Aid.** Commercial IGC. Updated in 2015 to include a Bumblebee 2 stereo optical camera module. Features a laser localisation aid, shown in Figure 2. Used for an extensive range of surgical applications.	CsI:Tl scintillator and a Hamamatsu H8500 flat-panel MA-PMT.	MA-PMT signals are multiplexed to 4 readout signals. Event position determined by a 2D polynomial model, parameterised by a least-squares fit of known positions.	Pinhole (⌀2.5 mm)	5.4 @ 3 cm 7.3 @ 5 cm	~135.1 @ 3 cm 49.6 @ 5 cm 17.1 @ 5 cm	15.9 ^f^	80×90×150 ^g^	-	40×40 @ 3 cm
				Pinhole (⌀4 mm)	7 @ 3 cm 11.1 @ 5 cm 21 @ 15 cm	~270.3 @ 3 cm 105.0 @ 5 cm 39.2 @ 3 cm				
YamamotoCam [20,62,63]	Designed for small-animal scintigraphy and noted for intraoperative suitability. Unique scintillator architecture: a continuous scintillator, scored on the object-facing surface to produce fine pixelation.	Grooved GAGG:Ce scintillator coupled to a Hamamatsu H8900 PS-PMT.	The 6X and 6Y cross-plate PS-PMT anode signals are passed to weight-summing amplifiers before digital conversion, giving 4 readout signals.	Pinhole (⌀1 mm)	1.0 @ 1 cm @ 122 keV	21.4 @ 1 cm @ 122 keV ^h^	18.5 @ 122 keV	-	-	12×12 @ 1 cm
SURGEOSIGHT-I [17,42]	Designed for preoperative and intraoperative scintigraphy for SLNB and radioguided cancer surgery.	Pixelated CsI:Na scintillator array and a Hamamatsu H8500 flat-panel MA-PMT.	The 64 anode MA-PMT signals are multiplexed to give 4 readout signals before digitisation. Event positioning by a COG algorithm.	Parallel (LEGP)	~2.2 @ 0 cm 9.4 @ 10 cm	142	20.6	-	-	-
λ-Eye [63,64,65,66]	Designed to optimise imaging performance considering the sensitivity and spatial resolution requirements needed for axillary sentinel lymph mapping.	Collimator–aperture-matched pixelated CsI:Tl scintillator array with tungsten septa and a Hamamatsu R8900U-00-C12 PS-PMT.	6X + 6Y PS-PMT output multiplexed to four readout signals. Event positioning by COG algorithm.	Parallel (crystal– collimator structure)	2.2 @ 0.2 cm ~10 @ 5 cm	1500	36	40×40×70	~1	22×22
PGC [31,67]	**Ultra-portable** IGC with integrated display, ARM computing system, and battery allowing intraoperative imaging without additional equipment or cabling.	Collimator–aperture-matched pixelated CsI:Tl scintillator array with tungsten septa, and a 7×4 array of Hamamatsu S11828-3344M (4×4 SiPM) MPPCs.	MPPC output multiplexed to 4 readout signals. Event positioning by COG algorithm, implemented on the integrated computing system [67].	Parallel (crystal– collimator structure)	~2.6 @ 0 cm ~5.4 @ 3 cm	142	16.2 @ 122 keV	150×90×70	~1	101.4×57.2
HCGC [11,23,68,69,70,71]	**Hybrid** Development of the Mini Gamma Ray Camera, featuring co-aligned gamma–optical/near-infrared imaging. Used for multiple clinical scintigraphy applications, including thyroid imaging and lymphoscintigraphy.	Columnar CsI:Tl scintillator and a Teledyne e2V CCD97 back-illuminated EMCCD.	Custom CCD readout. Event position is determined frame-by-frame using a blob-detection algorithm with automatic scale selection.	Pinhole (⌀0.5 mm)	1.28 @ 1.3 cm	214 @ 0.3 cm	58	⌀103 ×211 ^d^	1.5 ^d^	40×40 @5 cm ^d^
PolitoCam [21,42,72,73,74,75]	**Hybrid** Dual-modality gamma–ultrasound device featuring matched FOVs. Gamma components based on an earlier IGC.	LaBr${}_{3}$ scintillator and a Hamamatsu H10966 flat-panel MA-PMT	64 MA-PMT readout channels by 4 FPGA readout boards. Event positioning by a position-weighted, modified COG algorithm.	Parallel (HR)	2.5 @ 2 cm ^i^	-	7.1	-	-	50 × 50
JungCam [76]	Designed to provide sub-millimetre intrinsic spatial resolution in a small-footprint device.	Collimator-matched pixelated GaGG:Ce scintillator array coupled to an MPPC.	The 64 MPPC output channels are multiplexed to 4 readout signals.	Diverging	3.2 @ 10 cm	59.9 @ 0 cm @ 122 keV 27.9 @ 4 cm @ 122 keV 8.6 @ 10 cm @ 122 keV	18.9	50×50×126	0.9	65×65 @ 10 cm
MediPROBE [7,8,9,16,77]	Under continuous development since 2009. Feature multiple available collimators, including coded aperture geometries, and multiple readout ASICs. Used preoperatively for sentinel lymph mapping.	CdTl:Cl semiconductor with finely pixelated Ohmic contacts coupled to a Medipix2 or Timepix CMOS readout ASIC.	128- or 256-channel readout, with values subject to 2 energy thresholds (Medipix devices) or spectroscopic (Timepix devices). Event positioning by pulse centroid location using short-exposure frames.	Pinhole (⌀0.35 mm)	1.09 @ 5.4 cm @ 60 keV	-	-	92×217×30	3.2 ^j^	6.2×6.2 −40×40 @ 5 cm
				Pinhole (⌀0.94 mm)	2.57 @ 4.5 cm @ 60 keV	-				
				Pinhole (⌀1.9 mm)	3.2 @ 2.5 cm 5.0 @ 5 cm 8.2 @ 10 cm	230 @ 2.6 cm 34.0 @ 5.6 cm 5.4 @ 9.6 cm				
				Coded aperture (⌀0.07 mm)	0.56 @ 5 cm @ 60 keV	-				
KoglerCam [24]	Adapted version of the PopovicCam used within the freehand-SPECT (fhSPECT) system. Used preoperatively for sentinel lymph mapping.	60 mm-thick pixelated NaI(Tl) scintillator array and PopovicCam detector.	PopovicCam readout.	Modular parallel (×1)	4.5 @ 0 cm ^k^ 11.0 @ 5 cm ^k^ 18.0 @ 10 cm ^k^	171.0 @ 10 cm	21.5	⌀75×41	1.4	63×63

^a^ Nominal value. ^b^ Mean value, calculated from x/y directional spatial resolutions [43]. ^c^ Estimated values from published figure [10]. ^d^ Previously unpublished values. ^e^ System spatial resolution of the MAGICS camera was also characterised using a Pb parallel collimator with ⌀1 mm apertures and 5 mm septal thicknesses with event position calculated using an iterative Levenberg—Marquard algorithm to fit a point-spread function model to the charge distribution measured by the SiPMs. These measurements ranged from 1.03–1.32 mm @ 122 keV across the device’s FOV [32]. ^f^ Gamma imaging module only. ^g^ Energy unknown. ^h^ Calculated from percentage sensitivities provided [20]. ^i^ Non-air scattering media present. ^j^ Weight with removable 5 mm-thickness Pb shielding attached (1.5 kg with shielding removed) [8]. ^k^ Estimated values from published figure [24]. LEHR: Low-Energy High Resolution, LEGP: Low-Energy General Purpose, LEHS: Low-Energy High Sensitivity, MEGP: Medium-Energy General Purpose, HR: High Resolution.

**Table 2:** Characteristics of parallel-hole collimators used in intraoperative gamma cameras.

Device	Collimator Name	Aperture Shape	Aperture Diameter (mm)	Septal Thickness (mm)	Aperture Length (mm)	Geometric Resolution (mm)	Geometric Efficiency	Material
TReCam [18,89]	LEHR	Hexagonal	1.5	0.23	15	6.8 @ 5 cm 12.0 @ 10 cm	$5.7\times 10^{-4}$	Pb
IPG 2 [38,41]	LEGP	Square	2.25	0.2	24	7.0 @ 5 cm 11.8 @ 10 cm	$6.2\times 10^{-4}$	W
CrystalCam [43,90]	LEHR	Square	2.16	0.3	22.58	7.1 @ 5 cm 12.0 @ 10 cm	$5.9\times 10^{-4}$	W
	LEHS	Square	2.04	0.42	11.15	11.7 @ 5 cm 21.3 @ 10 cm	$2.0\times 10^{-3}$	W
	MEGP	Circular	1.5	0.96	11.5	8.5 @ 5 cm 15.4 @ 10 cm	$4.10\times 10^{-4}$	Pb
PopovicCam [10]	×1 Modular collimator	Square	0.6	0.4	5.5	7.4 @ 5 cm 14.1 @ 10 cm	$5.3\times 10^{-4}$	W-polymer composite ^a^
	×2 Modular collimator	Square	0.6	0.4	11	3.6 @ 5 cm 6.6 @ 10 cm	$1.0\times 10^{-4}$	W-polymer composite ^a^
GoertzenCam [52]	LEHR	-	1.2	0.2	23	-	-	-
MAGICS [32]	-	-	-	-	15	-	-	Pb
SURGEOSIGHT-I [17]	LEGP	Hexagonal	1.2	0.2	18	4.7 @ 5 cm 8.2 @ 10 cm	$2.40\times 10^{-4}$	Pb
λ-Eye [64]	crystal –collimator structure	Square	2	0.2	24	11.7 @ 5 cm ^b^ 21.5 @ 10 cm ^b^	$2.5\times 10^{-3}$ ^b^	Pb
PGC [31]	-	Square	2.4	0.2	24	7.5 @ 5 cm 12.6 @ 10 cm	$7.1\times 10^{-4}$	W
PolitoCam [91]	HR	Hexagonal	1	0.2	18	3.9 @ 5 cm 6.8 @ 10 cm	$1.6\times 10^{-4}$	Pb

^a^ Measured linear attenuation coefficient value of 18.9 cm${}^{-1}$ used [10]. ^b^ The use of a crystal–collimator structure results in the projection on the crystal face occurring at a distance lower than the aperture length. Instead, values calculated were for a collimator thickness of 11 mm, i.e., the collimator thickness before the crystal surface is reached [64].

**Table 3:** Characteristics of pinhole collimators used in intraoperative gamma cameras.

Device	Aperture Diameter (mm)	Acceptance Angle (${}^{\circ }$)	Thickness (mm)	Collimator–Detector Distance (mm)	Geometric Resolution (mm)	Geometric Efficiency	Material
Sentinella 102 [19]	2.5	-	-	-	-	-	Pb
	4	-	-	-	-	-	Pb
YamamotoCam [20]	0.5	-	-	18	-	-	-
MediPROBE_*Medipix2ASIC*_ [8,9,16,92]	0.35	90	13	18	2.04 @ 5 cm 3.55 @ 10 cm	$1.2\times 10^{-5}$ @ 5 cm $2.9\times 10^{-6}$ @ 10 cm	W
	0.94	90	4	18	4.27 @ 5 cm 7.41 @ 10 cm	$3.9\times 10^{-5}$ @ 5 cm $9.7\times 10^{-6}$ @ 10 cm	W
	1.9	90	4	25	6.27 @ 5 cm 10.45 @ 10 cm	$1.2\times 10^{-4}$ @ 5 cm $3.0\times 10^{-5}$ @ 10 cm	W
HCGC [68]	0.5	60	6	10	3.66 @ 5 cm 6.71 @ 10 cm	$1.1\times 10^{-5}$ @ 5 cm $2.9\times 10^{-6}$ @ 10 cm	W
	1	60	6	10	6.66 @ 5 cm 12.21 @ 10 cm	$3.4\times 10^{-5}$ @ 5 cm $8.6\times 10^{-6}$ @ 10 cm	W

**Table 4:** Characteristics of diverging collimators used in intraoperative gamma cameras.

		Object Side		Detector Side				
Device	Aperture Shape	Aperture Diameter (mm)	Septal Thickness (mm)	Aperture Diameter (mm)	Septal Thickness (mm)	Thickness (mm)	Focal Distance (mm)	Material
JungCam [76]	Square	0.7	0.35	0.7	0.1	20	65.5	WC

**Table 5:** Characteristics of coded-aperture collimators used in intraoperative gamma cameras.

Device	Design	Matrix	Aperture Shape	Aperture Diameter (mm)	Acceptance Angle (${}^{\circ }$)	Aperture Number	Thickness (mm)	Material
MediPROBE [9,77]	NTHT-MURA	62 × 62	Round	0.08	180	480	0.11	W
	NTHT-MURA	62 × 62	Round	0.07	180	480	0.08	W

**Table 6:** Architectures of pixelated-scintillator-array detector intraoperative gamma cameras.

Device	Architecture	Detector Dimensions (mm^3^)	Pixel Size (mm^3^)	Pixel Matrix	Total Readout Area (mm^2^)	Readout Pixel Size (mm^2^)	Readout Pixel Pitch (mm)	Readout Layout
IPG 2 [38,41,42]	Pixelated CsI:Tl + PS-PMT	44.1×44.1×5	2.25×2.25×5	18×18	49×49	5.8×5.8	6.08 ^a^	8×8
GoertzenCam [52,102]	Pixelated CsI:Tl + MPPC	13.2×13.2×5	3.3×3.3×5	4×4	13.4×13.4	3.16×3.16	3.36	4×4
YamamotoCam [20,62,63]	Grooved GAGG:Ce + PS-PMT	20×20×1	0.2×0.2×0.9	80×80 ^b^	23.5×23.5	N/A	N/A	6X + 6Y cross-plate
SURGEOSIGHT-I [17,42]	Pixelated CsI:Tl + PS-PMT	51.4×51.4×5	1×1×5	43×43	49×49	5.8×5.8	6.08 ^a^	8×8
λ-Eye [63,64]	Pixelated CsI:Tl + PS-PMT	20.8×0.8	1.9×1.9×5	10×10	23.5×23.5	N/A	N/A	6X + 6Y cross-plate
PGC [31,67]	Pixelated CsI:Tl + MPPC	101.1×56.9×5.5	2.3×2.3×5.5	39×22	102.2×58.4	3×3	-	28×16
JungCam [76,103,104]	Pixelated GAGG:Ce + MPPC	25.1×25.1×4 ^c^	0.7×0.7×3.5	29×29	25.8×25.8	3×3	3.2	8×8
KoglerCam [10,24]	Pixelated NaI:Tl + MPPC	62.25×62.25×60	2.25×2.25×60	25×25	~281	3×3	6	8×8, bounded by 1×4 arrays

^a^ At array centre. ^b^ Pixels connected by continuous 0.1 mm-thickness GAGG:Ce at detector side. ^c^ Includes volume of BaSO${}_{4}$ reflector material.

**Table 7:** Architecture properties of continuous-crystal-scintillator detector intraoperative gamma cameras.

Device	Architecture	Detector Dimensions (mm^3^)	Total Readout Area (mm^2^)	Readout Pixel Size (mm^2^)	Readout Pixel Pitch (mm)	Readout Layout
TReCam [18,35]	LaBr${}_{3}$:Ce + PS-PMT	- × - × 5	49×49	2.8×2.8	3.04 ^a^	16×16
PopovicCam [10]	LaBr${}_{3}$:Ce + MPPC	~296×6	~321	3×3	6	8×8, bounded by 1×4 arrays
MAGICS [32,53]	LaBr${}_{3}$:Ce + MPPC	51×51×5	53×54	3×3	-	16×16
Sentinella 102 [22,42,58]	CsI:Na + PS-PMT	40×40×4	49×49	5.8×5.8	6.08 ^a^	8×8
HCGC [11,70]	Columnar CsI:Tl + EM-CCD	- × - × 0.6	8.19×8.19	0.016×0.016	-	512×512
PolitoCam [21,42]	LaBr${}_{3}$:Ce + PS-PMT	50×50×4	49×49	5.8×5.8	6.08 ^a^	8×8

^a^ At array centre.

**Table 8:** Architecture of semiconductor detector intraoperative gamma camera devices.

Device	Architecture	Detector Dimensions (mm${}^{3}$)	Active Area (mm${}^{2}$)	Anode Pixel Pad Size (mm${}^{2}$)	Anode Pixel Pitch (mm)	Anode Matrix
CrystalCam [43,45,48,49]	CdZnTe + x2 XAIM ASICs	39 × 39 × 5	-	1.86 × 1.86	2.46 ^a^	16 × 16
MediPROBE_*Medipix2ASIC*_ [7]	CdTe:Cl + Medipix2 ASIC	- × - × 1	14.08 × 14.08	0.045 × 0.045	0.055	256 × 256
MediPROBE_*TimepixASIC*_ [7,9,114]	CdTe:Cl + Timepix ASIC	- × - ×t 1	14.08 × 14.08	0.045 × 0.045	0.11	128 × 128

^a^ At array centre.

## Data Availability

Not applicable.
